# The impact of faith-based organizations on maternal and child health care outcomes in Africa: taking stock of research evidence

**DOI:** 10.11604/pamj.2022.43.168.32983

**Published:** 2022-12-05

**Authors:** Jeannine Uwimana Nicol, Chinwe Juliana Iwu-Jaja, Lynn Hendricks, Peter Nyasulu, Taryn Young

**Affiliations:** 1 Center for Evidence Based Health Care, Division of Epidemiology and Biostatistics, Department of Global Health, Faculty of Medicine and Health Sciences, Stellenbosch University, Cape Town, South Africa,; 2 School of Public Health, College of Medicine and Health Sciences, University of Rwanda, Kicukiro, Kigali, Rwanda,; 3 Social Research Methodology Group, Faculty of Social Sciences, Katholieke Universiteit (KU) Leuven, Leuven, Belgium

**Keywords:** Faith-based organizations, maternal and child health, overview of reviews, Africa

## Abstract

This evidence synthesis aimed at assessing the effectiveness of Faith-Based Organisations (FBOs) on Maternal and Child Health (MCH) outcomes; and explore the perceptions and experiences of the users and providers of MCH services delivered by FBOs in Africa. This review considered studies from African countries only. Both reviews and primary studies focusing on MCH services provided by FBOs were considered. Quantitative, qualitative, and mixed methods reviews were included with no restriction on the date and language. Primary outcomes included maternal mortality ratio, neonatal mortality, infant mortality, child mortality, quality of care, views, experiences, and perceptions of users of FBOs. We searched up to November 2020 in the Joanna Briggs Institute (JBI) Database of Systematic Reviews and Implementation Reports, the Cochrane Database of Systematic Reviews, Database of Abstracts of Reviews of Effects, PROSPERO register, PDQ-evidence, Health Systems Evidence, CINAHL, EMBASE, and PubMed. We searched references cited by similar studies that may be potentially eligible for inclusion. We then updated the search for primary studies from December 2009 - October 2020. One systematic review and six primary studies met the eligibility criteria for inclusion. Methodological quality varied. These observational and qualitative studies found that FBOs offered the following MCH services - training of healthcare workers, obstetric services, health promotion, sexual education, immunization services, and intermittent preventive therapy for malaria. Maternal and Child Health (MCH) services provided by FBO suggest a reduction in maternal morbidity and mortality. Increased uptake of maternal healthcare services, and increased satisfaction were reported by users of care. However, costs of providing these services varied across the studies and users. This review shows that FBOs play an important role in improving access and delivery of MCH services and have the potential of strengthening the health system at large. Rigorous research is needed to ascertain the effectiveness of FBO-based interventions in strengthening the health systems in Africa.

## Introduction

According to the World Health Organization (WHO), maternal conditions are the leading causes of death and disability in low- and middle-income countries (LMICs) [1]. The World Health Organization (WHO) African region bore the highest burden of maternal deaths in 2015, with almost two-thirds occurring in the region [2]. Similarly, about 5.4 million children under age five died in 2017 with 74% occurring in Africa [1]. Almost all these deaths occur amongst the poorest and most disadvantaged population groups [1]. Kruk *et al*. evaluated the quality of care available to people in LMICs across a range of health needs, based on the Sustainable Development Goals (SDGs), and found that mothers and children receive less than half of recommended clinical actions in a typical preventive or curative visit [3]. To achieve tangible progress in global goals like the SDGs, a multi-sectoral approach to strengthening the health system is needed [4].

The complex social, political, environmental and economic determinants of health calls on health systems to look beyond the provision of care by a single entity such as the public healthcare services [5]. A private-public-partnership (PPP) such as Faith-Based Organizations (FBOs) can strengthen the health system and advance the progress to achieving the SDGs in sub-Saharan Africa. The literature highlights the importance of active engagement of FBOs in health programs and their role in improving equitable access to healthcare in LMICs [6]. Additionally, DeHaven *et al*. [7] argue that FBOs and churches are institutions that are known in their community and tend to succeed because of the trust that the community has for this institutions, and when outside healthcare professionals cannot. In most countries in sub-Saharan Africa, FBOs have been active in health and other developmental sectors for decades with an account of over 60% of health sector interventions [8-10].

An FBO is defined as an “organization, with or without non-profit status that provides social services and is either motivated by religious faith or belief or have affiliations with religious groups” [11]. More recently, FBOs have been described as “faith-based civil society organizations, informal faith-based programmes, initiatives and community-based organizations, larger national and international non-governmental organizations, congregations such as places of worship, religious leaders, faith-based healthcare facilities, and denominational and interdenominational health networks such as the Christian Health Associations” [12]. These organizations deliver between 30-70% of health services in LMICs [5,11]. In many countries in Africa, FBOs have been shown to be providing primary, secondary, and tertiary care to mostly poor populations [5,13], and have a significant influence on the health outcomes and health-seeking behaviour of users [5,12,13].

A number of published studies on the role and contribution of FBOs on maternal, newborn, and child health (MNCH), HIV/AIDS and other health issues have focused on understanding the different roles of FBOs in healthcare delivery, their reach, costs, and satisfaction from beneficiaries [5,12,14]. Considering the pressing need for LMICs to accelerate the achievement of MNCH targets as stipulated in the SDG and the potential contribution of FBOs in providing health care services in LMICs, particularly in Africa, there is a need to get a better understanding on how FBOs could contribute. We took a systematic approach to summarize the evidence on FBOs involvement in MCH programs in Africa, taking into consideration factors such as type of FBOs, context and type of services provided and reported outcomes in order to provide a catalogue of evidence on the effect and the contribution of FBOs in providing MCH related services. We aimed to synthesize research evidence on FBOs providing MCH programs including HIV in Africa - to examine and describe the research evidence on the effectiveness of FBOs on MCH outcomes in Africa; and to explore the perceptions and experiences of the recipients and providers of MCH services, delivered by FBOs in Africa.

## Methods

The protocol was registered in the International Prospective Register of Systematic Reviews (PROSPERO): CRD 42020153300 [15]. We conducted this evidence synthesis in two stages. Firstly, we took stock of existing systematic reviews and secondly searched for primary studies conducted outside of the date range of the systematic reviews identified.

**Criteria used to consider studies for inclusion:** we included systematic reviews and primary studies (quantitative, qualitative, and mixed method studies published outside of the date range of identified systematic reviews) on MCH services provided by FBOs. We considered any level of care and facilities providing either maternal or child services or both by any health provider regardless of cadres. The comparison was of other types of organizations or health care services providers that are not faith-based such as government-owned healthcare facilities and non-governmental organizations. We defined maternal health as the health of women during pregnancy, childbirth, and postpartum (the first 42 days after delivery) [16]. Child health refers to the health of children younger than five years (perinatal <7 days old, neonate <29 days old, child <5 years of age) [16]. MCH services included antenatal interventions, intrapartum interventions, newborn and neonatal interventions, management of birth-related complications, and child health interventions. Primary outcomes included maternal mortality ratio, neonatal mortality, infant mortality, child mortality (under five years of age), views, experiences, perceptions and quality of care.

Secondary outcomes included utilization of MCH services, the proportion of live births, percentage of births attended by skilled health personnel, uptake of intermittent preventive treatment (IPT) for malaria, the proportion of HIV pregnant women on prevention of mother to child transmission (PMTCT), immunization coverage, maternal and child morbidity, stunted children, the proportion of children receiving treatment of childhood infectious diseases, like pneumonia, cost-effectiveness, access to care, client´s satisfaction, quality of care and continuity of care as reported in the studies. Systematic reviews are defined as reviews with the following characteristics as described by Moher *et al*. 2015 [17]: clear defined objectives with an explicit reproducible methodology; a systematic search that attempts to identify all studies that would meet the eligibility criteria; assessment of the validity of the findings of the included studies (e.g. assessment of the risk of bias and confidence in cumulative estimates); systematic presentation, and synthesis of characteristics and findings of the included studies. Both quantitative, qualitative, and mixed methods reviews were considered. Reviews that incorporated only theoretical studies or published opinion as to their primary source of evidence were excluded.

**Search methods for identification of studies:** the first search used a comprehensive search strategy to identify systematic reviews. It was developed for PubMed and adapted for other databases, with the assistance of an information specialist (Annex 1). We did not apply any date or language restrictions. The following key words were used with key terms such as 'systematic' or 'meta-analysis' in the title or abstract fields: 'delivery of care/economics', 'organization and administration', 'community health centres', ‘faith-based organizations', 'faith-inspired interventions', 'hospitals/religious', 'quality of care', 'public-private partnerships', 'SDG3' and 'health services/research', 'faith-based facilities', 'religious programmes´, ‘local faith communities', 'humanitarian care', 'faith-based aid'. We searched up to November 2020 in the JBI Database of Systematic Reviews and Implementation Reports, the Cochrane Database of Systematic Reviews, Database of Abstracts of Reviews of Effects, PROSPERO register, PDQ-evidence, Health Systems Evidence [18], Current Index to Nursing and Allied Health Literature (CINAHL), EMBASE and PubMed. We searched references cited by other similar studies that may be potentially eligible for inclusion. We then searched for primary studies published in the period December 2009 to October 2020 (not covered in the included systematic review). A comprehensive search strategy was developed for PubMed and adapted for other databases, with the assistance of an information specialist (Annex 1). We searched in the JBI Database of Systematic Reviews and Implementation Reports, CINAHL, EMBASE, and PubMed.

**Selection of studies, data extraction, and management:** two authors (Chinwe Juliana Iwu-Jaja and Jeannine Uwimana Nicol) independently screened titles and abstracts of the records from the first search on Covidence^R^ software. The records were rated as 'or exclusion', 'for inclusion', or 'potentially eligible' accordingly.

The full text of systematic reviews judged as 'for inclusion' or 'potentially eligible' were then assessed against the inclusion criteria independently by the two authors. Disagreements were resolved be through discussions and when consensus was not reached, a third author (Taryn Young) was consulted for arbitration. In the second round of screening to identify recent primary studies (from 2009-2020) five reviewers (Jeannine Uwimana Nicol, Chinwe Juliana Iwu-Jaja, Lynn Hendricks, Peter Nyasulu and Taryn Young) independently screened titles and abstracts of the records in Covidence. The full text eligibility assessment was independently done by the same four reviewers. Disagreements were resolved through discussions and when consensus was not reached, two of the reviewers (Jeannine Uwimana Nicol and Chinwe Juliana Iwu-Jaja) resolved the conflicts. Data were extracted using a pre-defined form which included key characteristics of included studies such as first author name, year of publication; objectives, number of included studies; countries, characteristics of participants, study type, types of interventions, intervention intensities, setting, and context where the study was conducted, outcome assessed, instruments used to assess outcomes (including information regarding their reliability and validity); comparisons of performed and results. Additionally, extracted data included the phenomenon of interest (e.g. quality of care, satisfaction).

**Assessment of methodological quality, data analysis, and synthesis:** two authors (Chinwe Juliana Iwu-Jaja and Jeannine Uwimana Nicol) independently, and in duplicate, assessed the quality of systematic reviews using the AMSTAR 2 (A Measurement Tool to Assess Systematic Reviews) tool [19], qualitative evidence synthesis was assessed using the Joanna Briggs Institute (JBI) critical appraisal checklist for systematic reviews and research syntheses [20]. For primary studies, the JBI checklists for cross-sectional studies and economic evaluation studies [21] and the Critical Appraisal Skills Programme (CASP) [22] for qualitative studies were used. Discrepancies in the methodological quality assessments were resolved by discussion between two authors (Chinwe Juliana Iwu-Jaja and Jeannine Uwimana Nicol), and a third author (Taryn Young) was consulted to resolve any disagreement. Data were synthesized narratively. The Preferred Reporting Items for Systematic Reviews and Meta-Analysis (PRISMA) 2020 statement and flow diagram was used to summarize study selection [23] and guide reporting.

## Results

**Figure 1:**
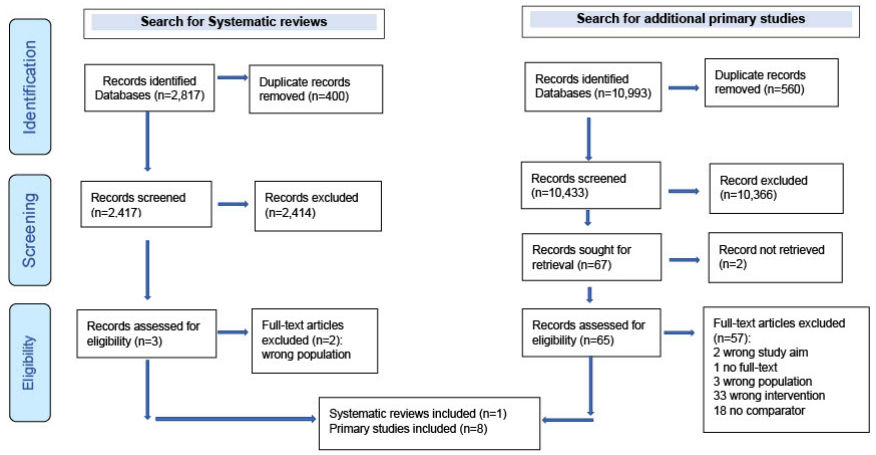
PRISMA flow diagram showing the process of study selection

**Results of the search:** a total of 13,810 article citations were identified through both stages of database searching, of which 12,844 were screened for titles and abstracts after de-duplication. These articles were a combination of primary studies and systematic reviews. After titles and abstracts screening, 64 studies (59 primary studies and three systematic review) were considered potentially eligible. We included one systematic review and eight primary studies (Figure 1).

**Characteristics of included studies:** the systematic review included six primary studies conducted in eight countries in Africa (1987-2007), while the eight additional primary studies were conducted from 2012-2020. The systematic review conducted by Widmer *et al*. [24] assessed the role of FBOs in the area of MCH in Africa. It included both qualitative and quantitative studies (three cross-sectional studies, one costing study, and one descriptive report). These studies were conducted in Nigeria, Tanzania, Ghana, Malawi, Uganda, Mozambique, and the Democratic Republic of the Congo (DRC). The additional primary studies were conducted in Tanzania, Rwanda, Cameroon, Ethiopia, Nigeria, Uganda, Kenya, Malawi, and the DRC [25-31]; these included five cross-sectional studies [25,29,31-33]; an economic evaluation [30] and two qualitative studies [26,28]. The characteristics of included studies are described in Table 1 and Table 2.

**Table 1:** characteristics of systematic reviews included

Study ID	Objective	Study design	Country and setting	Participants	Type of FBOs
**Systematic review - Widmer 2011, included 3 cross-sectional, 1 multimethod study, 1 economic study, 1 descriptive study**					
Lyun (1989)	An assessment of rural health programme on child and maternal care	Cross-sectional study	Rural community, Nigeria	Women of reproductive age (n=820)	Baptist Church (Ogbomoso Baptist Medical Centre)
Adetunji (1992)	An assessment of church-based obstetric care	Multi-method study comprising of cross-sectional study and in-depth interviews	Rural community, Nigeria	Women aged between 15 and 50 were surveyed while in-depth interviews were conducted on 25 of these women; 5 mission-trained midwives, 2 pastors, 5 government nurses, a local government dispenser, 5 patent medicine store operators, a private clinic owner and 6 male farmers	Christ Apostolic Church
Gilson (1995)	An assessment of the quality of Tanzanian primary health facilities	Cross-sectional study	Rural setting, Tanzania	40 government dispensaries (PHCs), 14 church dispensaries (PHCs), and 4 health centres	Catholic Church
Levin (2003)	An assessment of the cost of maternal health care services in 3 countries	Economic evaluation	Ghana, Malawi, Uganda	Public and mission hospitals and health centers	Mission hospitals and mission healthcare centres (denomination not specified)
Lindelöw (2003)	An assessment of public and private providers	Cross-sectional study	Rural and urban settings, Uganda	Medical doctors, nurses, nursing assistants	Catholic Medical Services, Uganda Protestant Medical Bureau, Uganda Muslim Medical Bureau, Seventh-day Adventist Church
Chand (2007)	Faith-based models for improving maternal and new-born health	Descriptive study	Uganda, Tanzania, Mozambique, the DRC	Nurses, doctors, birth attendants community heath volunteers religious leaders	The Uganda Protestant Medical, the Uganda Muslim Medical and the Uganda Catholic Medical Bureaus

FBOs: faith-based organizations; PHCs: primary health care

**Table 2:** characteristics of primary studies

Study ID	Objective	Study design	Country and setting	Participants	Type of FBOs
**Primary studies**					
Aristide (2020)	To construct a theoretical framework to understand the multi-dimensional factors impacting the decision to use FP in rural Tanzania	Qualitative	Tanzania rural villages, north-west	Women and men with a median age of 35 (IQR 26-46) years; church leaders	Protestant and Catholic churches
Mukamuyango (2020)	To explore potential obstacles and facilitators of an HIV-FP integrated program in by comparing long-acting reversible contraception (LARC) uptake in relation to rural/urban clinic location, Catholic/non-Catholic clinic affiliation, couple HIV sero-status and other participant characteristics	Quantitative cross-sectional	Rwanda peri-urban setting surrounding Kigali city	Heterosexual cohabiting couples not wanting to conceive in 2 years, non-pregnant women 21 and 40 years and men aged ≥ 21; planning to live in Kigali for at least 2 years; both partners fertile; women not using a LARC method	Catholic clinics
Mamo (2019)	To explore the role played by different actors in promoting ANC, childbirth and early PNC services, and mainly designed to inform a community-based information, education and communication intervention in rural Ethiopia	Qualitative	Ethiopia rural setting, south west region in Ethiopia	Health extension workers; Women and men development leaders; Women and men community leaders	Not specified
Adedine (2018)	To explore relationships between current use of a modern contraceptive method, exposure to family planning messages from religious leaders, and background characteristics	Quantitative cross-sectional	Nigeria 4 urban settings in Nigeria	Non-pregnant women aged 15 to 49 years	Christian and Muslim
Vossius (2014)	To analyze the cost-effectiveness of HBB at a faith-based Haydom Lutheran Hospital (HLH) in rural Tanzania	Quantitative economic evaluation	Tanzania, Northern Tanzania	Birth attendants	Lutheran Hospital
Vogel (2012)	To compare the delivery characteristics and outcomes in FBO’S using the WHO global survey on Maternal and Perinatal Health (WHOGS) to compare individual-level characteristics and outcomes of women and new-born delivering in those institutions	Quantitative cross-sectional	Uganda, Kenya and DRC the capital city and 2 randomly selected provinces	Mothers and new-borns during delivery and postpartum	Not specified
Barden-O’Fallon (2017)	To investigate the provision of family planning services by FBOs in three countries, Malawi, Kenya, and Haiti	Quantitative cross-sectional	Kenya, Malawi	Women of reproductive age	Catholics and Protestants
Oyugi (2018)	To evaluate perceived quality and satisfaction of the services under the output-based approach reproductive programme	Quantitative cross-sectional	Kenya rural setting	Women of reproductive age	Not specified

FBOs: faith-based organisations; FP: family planning; ANC: antenatal care; PNC: postnatal; HBB: helping babies breathe

The FBOs included Baptist Church, Christ Apostolic Church, Presbyterian Church, Catholic Church, Protestant Church, Catholic Church, Muslim organizations, Catholic Medical Services, Uganda Protestant Medical Bureau, Uganda Muslim Medical Bureau, the Christian Health Association of Malawi which includes Malawi Council of Churches and the Episcopal Conference of Malawi, the Kenya Conference of Catholic Bishops, Lutheran Church, and the Seventh-day Adventist Church. The services that were offered by these FBOs covered: capacity building: training of healthcare workers, community health workers, and volunteers in the community such as church/faith leaders, women leaders and others; health education and promotion; immunization services; obstetric and neonatal care services: births, management of neonates related complications, births and managing complications in newborns; sexual reproductive health and antenatal care: family planning services, antenatal care services including HIV testing for pregnant women, caesarian section and vaginal delivery, birth related complication and post-natal care up to 6 weeks after delivery; health education and promotion: hygiene, breastfeeding and immunization, family planning, counseling on family planning and HIV, malaria prevention including IPT, and Education on HIV prevention strategies; other primary health care services such as treatment of minor ailments, home-based care, community outreach programmes, among others; the reproductive services offered include safe motherhood (SMH) which comprise antenatal care (ANC) attendance, caesarian section and vaginal delivery, birth related complication and post-natal care up-to 6 weeks after delivery.

**The methodological quality of included studies:** the methodological quality of the systematic review by Widmer *et al*. [24] was limited due to lack of protocol *apriori*; there was no duplicate study selection and data extraction; no list of excluded studies; no risk of bias assessment; and no source of funding declared (Table 3).

**Table 3:** assessment of multiple systematic reviews (AMSTAR) 2 scores of Widmer *et al*.

AMSTAR 2 criteria	Yes	Partial yes	No	AMSTAR critical domains
Did the research questions and inclusion criteria for the review include the components of PICO?		X		
Did the report of the review contain an explicit statement that the review methods were established prior to the conduct of the review and did the report justify any significant deviations from the protocol?			X	Critical
Did the review authors explain their selection of the study designs for inclusion in the review?			X	
Did the review authors use a comprehensive literature search strategy?	X			
Did the review authors perform study selection in duplicate?	X			
Did the review authors perform data extraction in duplicate?	X			Critical
Did the review authors provide a list of excluded studies and justify the exclusions?			X	Critical
Did the review authors describe the included studies in adequate detail?			X	
Did the review authors use a satisfactory technique for assessing the risk of bias (RoB) in individual studies that were included in the review?			X	
Did the review authors report on the sources of funding for the studies included in the review?			X	Critical
If meta-analysis was performed, did the review authors use appropriate methods for statistical combination of results?			n/a	
If meta-analysis was performed, did the review authors assess the potential impact of RoB in individual studies on the results of the meta-analysis or other evidence synthesis?			n/a	
Did the review authors account for RoB in primary studies when interpreting/discussing the results of the review?			X	Critical
Did the review authors provide a satisfactory explanation for, and discussion of, any heterogeneity observed in the results of the review?			X	
If they performed quantitative synthesis, did the review authors carry out an adequate investigation of publication bias (small study bias) and discuss its likely impact on the results of the review?			n/a	
Did the review authors report any potential sources of conflict of interest, including any funding they received for conducting the review?	X			

n/a: not applicable

Four cross-sectional studies met six of the eight JBI criteria for quality [30-33] while the study by Mukamuyango *et al*. [25] met all the eight criteria of the JBI appraisal tool (Annex 2). The key methodological aspects that were not described include the identification of potential confounders and how these confounders were controlled in the analyses. The quality of two qualitative studies [26,28] was found to be methodologically adequate as they met all eight out of the CASP criteria (Annex 2). The methodological areas of concern included the lack of reflectivity of the researcher(s) and whether the data reached saturation. Vossius *et al*. [30] study complied with most of the questions of the JBI appraisal tool for economic evaluations. The areas of concern were the lack of description of the alternative intervention and the lack of relevant costs and outcomes for the alternative intervention. Also, there was no clear indication of the incremental costs, and not all issues of all concerns were taken into consideration while costing (e.g. training fee). The methodological quality appraisal of the qualitative and economic studies are presented as Annex 3 and Annex 4.

**Effects on FBOs on maternal and child health outcomes:** the key findings on MCH services provided by FBOs are presented in Table 4. Four cross-sectional studies included in the review and conducted in Nigeria, Tanzania, Ghana, Malawi, and Uganda, focused on MCH services related to health workers´ training, health promotion, community outreach (i.e. home visits), obstetric care, and quality of care provided by FBOs [34-37]. Seventy-four percent (74%) of women received education on maternal immunization, of which 69% received maternal immunization, and 27% received prenatal care from previously trained village health workers [34]. In addition, 40% of births took place in faith-based facilities, 43% of births occurred in government facilities, and 17% of births at home [35]. Women in Nigeria preferred the FBO-run services to the government facilities because of the perception that FBO run clinics were cleaner and had better outcomes [35]. In contrast, a study conducted in Uganda found no difference in services (including prenatal services) and quality of services offered by government and private for-profit and private not-for-profit, including FBOs [36]. However, the faith-based health facilities were reported to have better laboratory services, better working environment, and provided more affordable services accessible to the poor [36].

**Table 4:** key findings on maternal and child health services provided by FBOs

Services provided	Type of FBOs	Findings
Health education and promotion on MCH services	Muslim bureau; Catholic bureau; Seventh Adventist church; Protestant church	Increase in uptake of MCH services
Prenatal care	Muslim bureau; Catholic bureau; Protestant church; Lutheran church	Contribute to decrease in MCH mortality; increase in the uptake of ANC visits
Immunization of children	Muslim bureau; Catholic bureau; Lutheran church	Increase in immunization coverage
Sexual and Reproductive Health (family planning)	Baptist church; Catholic church; Protestants	Increase in the uptake of family planning; family planning services lower in FBOs than their counterparts
Obstetric care	Christ Apostolic church; Protestant church; mission hospitals (Catholic church mainly); Lutheran church	Contribute to decreased maternal and infant mortality; increase in early ANC visits
Maternal and childcare (e.g. Obstetric care, Maternal immunization and child immunization)	Baptist church; Christ Apostolic church; Catholic Church	Patients’ satisfaction, affordability and accessibility
PMTCT and HIV care	Catholic bureau; Protestant church	Contribute to prevention of mother to child transmission; increase in uptake of HIV testing and family planning
Other services (training of health professionals, Home based care (HBC))	Catholic church; Lutheran church; Protestant	Contribute to quality care and performance

FBOs: faith-based organisations; MCH: maternal and child health; PMTCT: prevention of mother to child transmission; ANC: antenatal care

When comparing government, FBOs, and private health facilities in terms of MCH services frequently rendered in the community, most government and FBO facilities provided obstetric services and prenatal care. The majority of FBOs (88.6%) and the government facilities (98.7%) offered prenatal care. There were higher immunization levels among women and children in the FBO facilities than government facilities offering similar services. Faith-Based Organizations (FBOs) had more supplies of health commodities than government facilities [38]. In addition, a cross-sectional study examining both PMTCT and family planning (FP) services by catholic and protestant churches in Rwanda found an increased uptake of these services [25]. A descriptive analysis that compared NGO and FBO-run institutions in Uganda, Kenya, and the DRC [31], found 22 FBO institutions delivered 11,594 women, compared to 20 government-run institutions delivering 25,825 women in the same countries and period. Infrastructure, obstetric services, diagnostic facilities, and anesthesiology at NGO/FBO institutions were comparable. A cross-sectional study conducted in Nigeria assessed exposure to religious leaders tailored scriptural family planning messages with contraceptive use. Among the non-pregnant women aged 15 to 49 years evaluated, there was a higher uptake of modern contraceptives among women with high exposure to messages (35.5%) than respondents in the low or medium exposure categories (14.5% and 24.5%, respectively). A higher contraceptive uptake was reported among women who had exposure to family planning messages from religious leaders compared to those with no exposure [29]. On the other hand, a multi-country study, showed that FBOs were less likely to provide family planning services than their counterparts. This study showed that in Kenya, 69% of FBOs provided these services compared to 97% and 83% of public and private facilities respectively. Also, FBOs provided low permanent or long-acting forms of contraceptives in Malawi (43%) and Kenya (29%) [32].

One of the included studies in the review [37] assessed the costs and quality of maternal services in different types of health facilities, including government hospitals, mission hospitals, and health centers. This study highlights no difference in the availability of drugs and equipment between government and FBO hospitals at the hospital level. However, at the health center level, availability of medicines and equipment and client satisfaction were predominately found in facilities run by FBOs in two of the countries. The quality of maternal services was also reported to be better in the FBO-run facilities than government facilities. The costs associated with obstetric complications were more in the public facilities in Malawi and Ghana. In Uganda, however, costs were higher in the FBO hospitals because of more staff and time put into service delivery. The additional cost-effectiveness study assessing the cost-effectiveness of training birth attendants found a decrease in neonatal mortality from 11.1 to 7.2 deaths per 1000 deliveries. These deliveries occurred in missionary hospitals in Tanzania. This intervention was cost-effective, with the cost per life saved was $233, while the cost per life gained was $4.21. The cost per disease-adjusted life year (DALY) averted ranged between $12- $23 [30]. Also, the quality of services provided by FBOs were perceived to be high in a study conducted in Kenya [33].

A descriptive report [39] included in the review covered Protestant churches, Catholic churches, and Muslim organizations in various African countries including Uganda, Tanzania, Mozambique, Malawi, and the DRC. This report showed an increase in uptake of IPT from 43% to 94% for the first course and from 26% to 76% in the second course in Uganda. Also, the hospital run by the Muslim organization in Uganda had lower maternal mortality compared to public hospitals. In Mozambique, volunteers were trained on hygiene, breastfeeding, and immunization. This led to a reported 50% reduction in child mortality in the FBO facilities. In this community, authors reported that religious leaders played a critical role in linking the FBOs with the community. In Tanzania, the report described an FBO which was run by a Muslim organization and was involved in offering obstetric services and managing complications in newborns. A congregation-based program was organized in Malawi in the year 2000 which involved churches or mosques or both. The program included health promotion activities among women groups as well as malaria prevention program. An evaluation survey of this program showed that most women (81%) were aware of the benefits of proper use of mosquito nets. In the DRC, a partnership between the Ministry of Health and FBOs showed an increase in uptake of prenatal services from 73.2% in 2001 to 86.2% in 2004; and an increase in birth assistants from 45% in 2001 to 64.3% in 2005 [39].

From the qualitative studies, Aristide *et al*. [26] highlighted the experiences of women who accessed family planning services in Tanzania and reported that the increased uptake of these services was influenced by support from family members and religious leaders. Similarly, the study in Ethiopia [28] highlighted how the continuous support from religious leaders coupled with the promotion of maternal health services (such as antenatal care) and child care services increased community members' trust and thus increasing the uptake of these services.

## Discussion

In this study, we synthesized research evidence on FBOs providing MCH programs including HIV in Africa. The findings provide important insights into the contribution and potential impact of FBOs in the delivery of MCH services in Africa. Improving the utilization of antenatal care services, such as getting more mothers to attend professional healthcare visits while pregnant, is critical to improving maternal and child health [40]. During the last few decades, there has been a growing interest in documenting the effectiveness of FBOs offering antenatal care, especially in low-income countries [41]. Our study revealed an increase in the utilization of prenatal services provided by FBOs, including IPT for malaria. A study conducted in Ghana reported a similar finding, where there was an increase in the overall utilization of health services offered by the FBOs [42]. Furthermore, the study, alongside another done in Burkina Faso, also reported significant gains in the areas of immunization and reduction in infant mortality [42,43] in line with findings of this review.

High cost of health services has been described as a major limitation to accessing care [43]. The cost of providing antenatal care has been shown to vary across studies [41] which is also in line with the findings from this review, where as in Malawi and Ghana, less costs were incurred in providing obstetric services to women. In contrast, in Uganda, more cost was incurred. A study conducted in Rwanda and Tanzania showed that the cost was more in the private facilities than in the FBOs [41,44]. Differences in costs have been attributed to differences in types of providers and ownerships of the health facilities as well as methods used to estimate these costs [38,44]. Although Orach [45] in his study on contracting health services through Uganda Catholic Medical Bureau, shows that healthcare services provided by Catholic Mission based health care facilities provided affordable services. It is imperative to have a robust body of knowledge on costing and cost-effectiveness of FBOs in delivering MCH services in Africa and other healthcare services in general.

Women preferred the services run by the FBOs to those by the government facilities [42]. These women´s perception was based the cleanness of health facilities run by FBOs and the better health outcomes associated to the quality of services rendered by these facilities. This is in line with the belief that FBOs are generally preferred by users [42]. Studies conducted in Ghana and Burkina Faso, for example, showed that people preferred the FBOs because of their high reputation. This high reputation was attributed to the quality of service [43,46] and the lower cost compared to other providers [43,45]. There is also an implicit trust and perception that the FBOs do not prioritize money and may likely not extort patients [43].

A study by Mwarey *et al*. [47] conducted in eight African countries (the DRC, Ghana, Lesotho, Malawi, Uganda, Tanzania, Zambia and Zimbabwe) also showed the satisfaction is beyond the maternal and health services but extends to other healthcare services [47]. Apart from the perception of good hygienic practices perceived by users of services provided by the FBOs, more reasons identified from other studies included their unique mode of operation, display of empathy and workforce motivation [42]. According to Olivier *et al*. patient satisfaction is so critical that it has been used to assess the quality of care and health-related outcomes and behaviors [42]. These perceptions could lead to increased utilization of services provided by the FBOs. Also, it´s imperative for policy makers and developmental partners involved in healthcare to invest in FBOs led facilities by maximizing the potential these FBOs have in improving MCH outcomes in Africa.

One systematic review and six additional primary studies met the inclusion criteria - consisting of cross-sectional, qualitative, economic evaluation and pilot studies. Methodological quality appraisal of these studies raised some concerns such non-reporting confounding factors and how these confounding factors were controlled (for cross-sectional studies); non reporting of the reflexivity of the authors/researchers (for quality studies); and lack of clarity on incremental costs and only the provider point of view was considered in the economic evaluation. It´s important to note that the scarcity of reviews and poor methodological quality of the included studies is not an isolated situation for the African continent, but it has been highlighted in other systematic reviews conducted among Latinos and Black Americans [7,48] Kagawa *et al*. [5], in their systematic and meta-analysis review on the scale of FBOs services in participation in healthcare delivery in developing countries, argue that the scarcity of data on the scale of FBOs in healthcare service delivery is mainly due to effects of political systems.

We followed a pre-specified protocol, conducted a comprehensive search, without language restrictions, covering various electronic databases, and we searched for on-going and unpublished systematic reviews. Two reviewers independently applied pre-defined eligibility criteria to select studies for inclusion, extracted data and evaluated the methodological quality of each included study. PRISMA 2020 reporting guidelines were followed [23]. Only observational, qualitative, and economic evaluation studies were included evidence on impact is limited. As the search strategy did not extensively include terms focusing on perceptions of beneficiaries and providers on services provided by FBOs, we might have missed some studies. Nonetheless, the included studies provide a landscape of evidence on the contribution, and perception, of FBOs on MCH programs on the African continent.

With regards to policy and public health implications, strengthening health systems in Africa through public private partnerships (PPP) such as FBOs, further research is needed to ascertain the effectiveness and impact of FBO based interventions on MCH outcomes and other global health programs. This will require capacity building of FBOs, partnerships of FBOs and academia/research agencies and increased funding to support a pipeline of evidence generation that will enhance informed decision making and the attainment of SDGs at large in developing countries. DeHaven *et al*. (2004) in their review assessing the effectiveness of FBO based health programs indicate that there is an urgent need for collaboration between FBOs and researchers to facilitate capacity building of FBOs in conducting research and routine health information systems to improve the quality of scientific papers produced. Additionally, Derose and Rodriguez [48] argue that means are needed to improve the frequency with which church-based health interventions or programs are evaluated, and the results of these evaluations are disseminated for informed decision making by policy makers.

## Conclusion

Faith-Based Organisations (FBOs) play an important role in health systems strengthening and their presence could contribute to MCH through the reduction in maternal mortality and morbidity, increased uptake of maternal health care services, increased satisfaction by users of care, provision of HIV/AIDS services and an overall improvement in health outcomes. Future research need to carefully consider the questions to be addressed and refine these, based on existing evidence to avoid unnecessary duplication [49,50]. Adherence to rigorous methodological approaches and good reporting practices [51] are important to ensure a contribution to evidence. There is a need for more rigorous studies to ascertain the effectiveness of FBO based interventions related to MCH. Thus, will enable policy makers in the health sector from the African continent in their decision-making process to develop national and regional health strategic plans to improve MCH outcomes through involvement of FBOs services.

**Funding statement:** this systematic review was supported by the funding from Stellenbosch University, Faculty of Medicine and Health Sciences-Early Career Research funding 2018. Award/grant number is not applicable. The funder did not have any role in the review process.

### What is known about this topic

- *There is high burden of maternal deaths in the African region which is common amongst the poorest and most disadvantaged population groups;*
- *Faith based organizations (FBOs) deliver between 30-70% of health services in low- and middle-income countries in Africa, including maternal and child health services;*
- *The role of the FBOs and their impact on maternal health outcomes in Africa need to be clearly understood*.

### What this study adds

- *Faith-Based Organisations (FBOs) play an important role in improving access and delivery of maternal and child health care services;*
- *Experiences of participants using services provided by FBO are variable;*
- *Further research is needed to ascertain the effectiveness of FBO-based interventions in improving maternal and child health outcomes and ultimately strengthening health systems in Africa*.

## Annexes

**Annex 1**: search strategy PUBMED (PDF-93 kb)

**Annex 2**: quality appraisal for cross-sectional studies (PDF-108 kb)

**Annex 3**: quality appraisal for qualitative studies (PDF-88 kb)

**Annex 4**: quality appraisal for economic evaluation studies (PDF-89 kb)
